# Interplay of the Gastric Pathogen *Helicobacter pylori* with Toll-Like Receptors

**DOI:** 10.1155/2015/192420

**Published:** 2015-04-06

**Authors:** Suneesh Kumar Pachathundikandi, Judith Lind, Nicole Tegtmeyer, Emad M. El-Omar, Steffen Backert

**Affiliations:** ^1^Department of Biology, Division of Microbiology, Friedrich Alexander University Erlangen-Nuremberg, Staudtstraße 5, 91058 Erlangen, Germany; ^2^School of Medicine & Dentistry, Institute of Medical Sciences, Division of Applied Medicine, Aberdeen University, Foresterhill, Aberdeen AB25 2ZD, UK

## Abstract

Toll-like receptors (TLRs) are crucial for pathogen recognition and downstream signaling to induce effective immunity. The gastric pathogen *Helicobacter pylori* is a paradigm of persistent bacterial infections and chronic inflammation in humans. The chronicity of inflammation during *H. pylori* infection is related to the manipulation of regulatory cytokines. In general, the early detection of *H. pylori* by TLRs and other pattern recognition receptors (PRRs) is believed to induce a regulatory cytokine or chemokine profile that eventually blocks the resolution of inflammation. *H. pylori* factors such as LPS, HSP-60, NapA, DNA, and RNA are reported in various studies to be recognized by specific TLRs. However, *H. pylori* flagellin evades the recognition of TLR5 by possessing a conserved N-terminal motif. Activation of TLRs and resulting signal transduction events lead to the production of pro- and anti-inflammatory mediators through activation of NF-*κ*B, MAP kinases, and IRF signaling pathways. The genetic polymorphisms of these important PRRs are also implicated in the varied outcome and disease progression. Hence, the interplay of TLRs and bacterial factors highlight the complexity of innate immune recognition and immune evasion as well as regulated processes in the progression of associated pathologies. Here we will review this important aspect of *H. pylori* infection.

## 1. Introduction 

The Gram-negative bacterium* H. pylori* is an extracellular pathogen infecting about 50% of the human world population. Infections with* H. pylori* can persist lifelong and are associated with chronic, often asymptomatic gastritis in all infected individuals, while some individuals can develop more severe gastric diseases such as peptic ulceration, MALT lymphoma, and gastric cancer [1, 2]. Disease progression is controlled by multiple factors such as genetic predisposition of the host, bacterial genotype, and environmental constraints [2–5]. Clinical* H. pylori* isolates are highly diverse both in their genetic polymorphisms and potential to induce pathogenicity. Myriads of bacterial factors have been associated with* H. pylori* pathogenesis. Well-known pathogenicity-associated mechanisms include urease-mediated neutralization of pH, flagella-driven bacterial motility, shedding of outer-membrane vesicles, secretion of proteases (such as HtrA) in the extracellular space, and peptidoglycan-dependent immune responses [6–10]. However, the two major* H. pylori* virulence factors are the vacuolating cytotoxin (VacA) and the CagA protein encoded by the cytotoxin-associated genes pathogenicity island (*cag*PAI). VacA is secreted in the culture medium and can induce multiple responses such as pore formation in the host cell membrane, modification of endolysosomal trafficking, cellular vacuolation, immune cell inhibition, and apoptosis, whereas the* cag*PAI represents a type IV secretion system for delivery of CagA into the host cell [11–14]. In addition,* H. pylori* express various typical surface adhesins, which permit the tight interaction of the bacteria with host target cells. The* Helicobacter* outer membrane porin (Hop) class of factors plays an important role in this context, comprising several well-described proteins including BabA, SabA, AlpA/B, OipA, HopI, HopQ, and HopZ [4, 15, 16]. Interestingly,* H. pylori* targets various host surface structures such as carbohydrates, phosphatidylserine, heparin sulfate, cholesterol, sphingomyelin, and other lipids as well as a broad range of host protein receptors [15, 17, 18]. Here we review the various molecular strategies of* H. pylori* to hijack a specific class of host protein receptors, the toll-like receptors (TLRs). We focus on the identified TLR family members and bacterial factors but also discuss several downstream signaling cascades, which are a crucial part of the host immune system.

## 2. Composition and Function of TLRs

**Figure 1:**
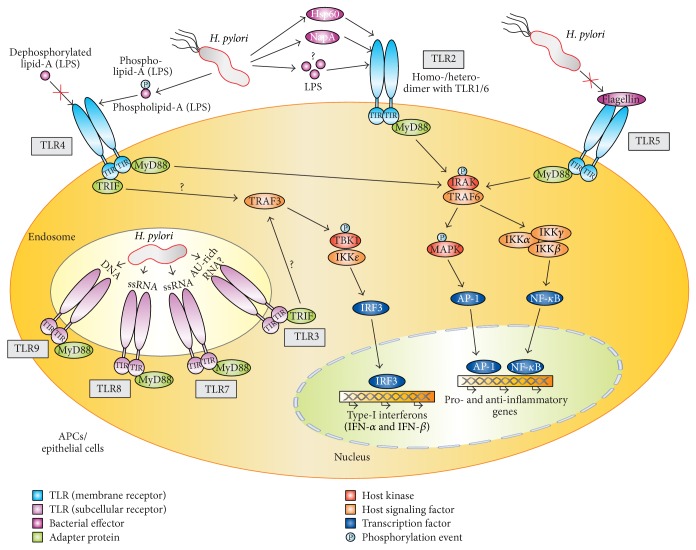
*H. pylori* mediate crosstalk with Toll-like receptors (TLRs) to manipulate signaling in innate immunity. TLRs constitute a group of cell surface and subcellular transmembrane receptors in antigen-presenting cells (APCs) and epithelial cells.* H. pylori* can interact with at least five TLR members (TLR2, TLR4, TLR5, TLR8, and TLR9) in various ways as indicated. TLRs are composed of a leucine-rich repeat-containing ectodomain, a transmembrane region and an intracellular tail with the TIR domain.* H. pylori* encodes various factors that have evolved either to target or to evade detection by the TLRs.* H. pylori* LPS, phosphorylated lipid A of LPS, HSP-60, NapA, DNA, and RNA are reported in various studies to be recognized by specific TLRs as shown. However, nonphosphorylated lipid A and flagellin evade the recognition by TLR4 and TLR5, respectively. The TIR domain in TLRs has a crucial role in adapter protein recruitment and the activation of downstream signaling cascades. TLR activity is initiated by PAMP-induced receptor dimerization and TIR engagement with the adapter proteins MyD88 or TRIF as indicated. Binding between a given TLR and MyD88 results in the recruitment of members from the IRAK kinase family. IRAK members are sequentially phosphorylated and dissociated from MyD88. This results in the activation of TRAF6, which in turn stimulates signaling through MAP kinases and IKK complex leading to the activation of transcription factors NF-*κ*B and AP-1 and the production of pro- and anti-inflammatory mediators. The adapter protein TRIF participates in the MyD88-independent TLR4 pathway as well as in the TLR3 signaling cascade. TRAF3 is recruited for the TRIF-mediated pathway mediating the production of type-I interferons and in some cases anti-inflammatory cytokine IL-10 [23, 24]. Endosomal TLR-mediated signaling leads to the induction of type-I interferons by the engagement of the transcription factor IRF as indicated. Question marks indicate activities/pathways which are not fully clear and require further investigation. For more details, see text.

TLRs constitute a group of cell surface and subcellular transmembrane proteins, which are expressed on cells of the host immune system including macrophages and dendritic cells (DCs) but are also present on the gastrointestinal epithelium and other nonimmune cells [19–21]. TLRs belong to the class of pattern recognition receptors (PRR) of the host innate immune system. These germ line encoded type I transmembrane glycosylated protein receptors are composed of an ectodomain with leucine-rich repeats (LRR), transmembrane region, and intracytoplasmic Toll/IL-1 receptor (TIR) domain [20–22]. TLRs act as sensors where they recognize microbial pathogens of bacterial, viral, fungal, and protozoan origin. A wide variety of ligands can bind to different TLRs to induce downstream signaling. The ligands are called pathogen associated molecular patterns (PAMPs) and include a broad array of microbial molecules build-up of proteins, nucleic acids, lipids, or synthetic chemicals. Upon activation by microbial factors, TLRs trigger the coordinated expression of host genes involved in specific signaling cascades for the regulation of innate and adaptive immunity, tissue repair, and regeneration processes [19, 20]. By binding of microbial ligands to a given TLR, there is activation of signaling transduction pathways involving the TIR domain and binding to cytoplasmic adaptor molecules including myeloid differentiation factor 88 (MyD88), TIR domain-containing protein (TIRAP), TIR domain-containing adaptor inducing interferon-beta (TRIF), and TIR domain-containing adaptor inducing interferon-*β*-related adaptor molecule (TRAM). This complex has been described to activate two main signaling pathways, the MyD88-dependent (applied by most TLRs except TLR3) and the MyD88-independent TRAM/TRIF cascade (applied by TLR3 and some signals of TLR4) [20–22]. The MyD88-dependent pathway signals through a cascade of interleukin-1 receptor associated kinase (IRAK), tumor necrosis factor receptor associated factor 6 (TRAF6), and transforming growth factor-beta-activated kinase 1 (TAK1) and activates transcription factor nuclear factor kappa B (NF-*κ*B) and its translocation from the cytoplasm to the nucleus as well as c-jun N-terminal kinase (JNK) and p38-mediated activator protein 1 (AP-1) stimulation [19–21]. NF-*κ*B and AP-1 can then bind to the promoter region of a variety of immune and inflammatory genes resulting in the transcription of proinflammatory and anti-inflammatory cytokines, including tumor necrosis factor- (TNF-) *α* and interleukin-6 (IL-6). The MyD88-independent pathway uses TRAM/TRIF adaptor proteins for initiating signaling, which leads to the cascade involving TRAF3, inhibitor of nuclear factor kappa-B kinase subunit epsilon (IKK*ε*), and TANK binding kinase 1 (TBK-1) to the production of type-1 interferons [19–21]. In addition, the TRIF pathway was reported to activate NF-*κ*B through a cascade involving receptor interacting protein 1 (RIP1) and TAK1. In this manner, TLRs regulate the production of cytokines, opsonization, coagulation cascades, complement activation, and upregulation of costimulatory molecules on antigen presenting cells. However, exact role of TLRs in* H. pylori* infection is highly controversial in the literature and needs to be reviewed. The various reported interactions with TLR2, TLR4, TLR5, TLR8, and TLR9 as well as some identified bacterial interaction partners and resulting downstream targets are summarized in Table 1. An overall model for major TLR activities and proposed signaling strategies exploited by* H. pylori* is presented in Figure 1.

## 3. Interaction of* H. pylori* with TLRs and Downstream Signaling

### 3.1. TLR2 in* H. pylori* Infection

The actual* H. pylori* ligand(s) of TLR2 are so far elusive and high efforts are made to detect the relevant contributors in activation. In general, TLR2 is able to recognize various different PAMPs, most importantly bacterial lipoproteins, lipoteichoic acid, or peptidoglycan. TLR2 forms heterodimers with either TLR1 or TLR6 to build a specific PRR able to recognize these different PAMPs [21, 25]. The surface expressed TLR2 receptor has so far been detected in intestinal and gastric epithelial cells and is known to be important in activating the inflammation system [26–28]. An important model cell system is cultured human embryonic kidney 293 (HEK293), endogenously not expressing most of the TLRs. These cells are therefore often used to study TLR involvement in infections by stably transfecting them with different reporter systems such as luciferase, SEAP (secreted embryonic alkaline phosphatase), and others. For example, Smith and coworkers investigated the expression of proinflammatory signals of various* H. pylori* strains on HEK293 cells that were cotransfected with TLR2, TLR5, or TLR4 and an NF-*κ*B luciferase reporter [28].* H. pylori* activated NF-*κ*B primarily through TLR2, but not TLR4, in stably transfected HEK293 and MKN45 gastric epithelial cell lines and induced chemokine expression (IL-8, MIP3*α*, and GRO*α*) through these TLRs. In these cells, TLR2 induction resulted in highly enhanced expression of chemokines as compared to TLR5. As control, expression of dominant-negative TLR2 mutants of gastric epithelial cell lines showed reduced activation by* H. pylori* and suggested that* H. pylori* lipopolysaccharide (LPS) is a TLR2 ligand [28]. However, it was reported that whole* H. pylori* cells activated TLR2 in transfected HEK293 cells, while LPS recognition appeared to be mediated by TLR4 [29]. In addition,* cag*A-positive strains were found to be more potent in activation of TLR2 than isogenic* cag*A mutant strains, suggesting the involvement of CagA or associated genes of the* cag*PAI in signaling and cytokine secretion. Moreover,* H. pylori* was found to induce IL-8 secretion in a TLR2-dependent manner rather than TLR4. Macrophages of TLR4-deficient mice (TLR4^−/−^) were also still able to produce strong cytokine and chemokine secretion (IL-6 and MCP-1), while macrophages of TLR2-deficient mice were not responsive during* H. pylori* infection [29]. In DCs derived from mice, the response to bacterial lysate also appeared to be induced via TLR2 and only to a minor extent by TLR4. On the other hand, the production of anti-inflammatory cytokine IL-10 was induced by* H. pylori* in DCs and that is completely abrogated in TLR2^−/−^ mice derived cells [30]. The production of IL-10 through activation of TLR2 could be linked to the observation that TLR2^−/−^ mice clear* H. pylori* infection more efficiently than infected wild-type animals [31]. Moreover, it was also shown that TLR2/MyD88 signaling induced by* H. pylori* in mouse macrophages led to the secretion of proinflammatory cytokines such as IL-6 and IL-1*β* [32]. A study on human neutrophils infected with* H. pylori* showed an induced expression of TLR2 and TLR4. In addition, an increased expression of IL-8, IL-10, IL-1*β*, and TNF-*α* was also found in neutrophils, while only IL-8 and IL-10 expression was reduced by using function-blocking anti-TLR2 and/or anti-TLR4 antibodies [33]. We have shown that* H. pylori* is able to induce TLR2 expression in monocytes (THP1 cells) and HEK293 epithelial cells stably transfected with TLR2. This also resulted in significant production and secretion of the proinflammatory cytokine TNF*α* and chemokine IL-8 in these cells [34]. It was also reported that highly purified* H. pylori* LPS can be detected through TLR2 (but not TLR4) and the TLR2 induction was dependent on the coexpression of TLR1 or TLR6 in HEK293 transfected luciferase reporter cell lines. Among TLR2/TLR6 and TLR2/TLR1 heterodimers, TLR2/TLR1 heterodimer was preferentially recognized by* H. pylori* LPS over TLR2/TLR6 heterodimer [35]. Nevertheless, there are also studies showing opposing activation and stimulation results. For example, Viala and coworkers used HEK293 cells (not expressing TLR2) which resulted in NF-*κ*B activation due to NOD1 recognition of peptidoglycan and thus cytokine induction irrespective of TLR expression [6]. In addition, other antigens of* H. pylori* have also been reported to trigger TLR2 activation. Addition of recombinant heat shock protein 60 (HSP60) of* H. pylori* to KATO-III gastric epithelial cells was shown to be able to trigger NF-*κ*B activation and upregulated expression of IL-8 [36]. In accordance with this study, Zhao and colleagues have shown that HSP60 induced the secretion of IL-8 in NOMO1 monocytic cells through activation of mitogen activated protein (MAP) kinases [37]. In particular, the extracellular signal regulated kinase (ERK) and p38 MAP kinase pathways are involved and this was abrogated in the presence of anti-TLR2 function-blocking antibodies or TLR2 siRNA [37]. The neutrophil activating protein (NAP) of* H. pylori* was also reported to activate TLR2 and induced the production of Th1 inflammatory cytokines such as IL-12 and IL-23 [38]. Although some studies are highly controversial, the above data clearly affirm an important role of TLR2 in* H. pylori* recognition and induction of proinflammatory changes. However, there should be further systematic analysis of these factors and binding mechanisms to TLR2, which may help us to better understand the varied roles of this important TLR during* H. pylori* infection.

### 3.2. TLR4 in* H. pylori* Infection

TLR4 was the first identified receptor as an ortholog of drosophila toll in mammals [57]. Further studies have identified LPS of bacteria as the ligand for TLR4 and binding of this ligand was dependent on the cofactors cluster of differentiation 14 (CD14) and myeloid differentiation factor 2 (MD2) [58–60]. The classical initial immune response induced by bacterial infection is generally mediated by macrophages and derives from the activation of TLR4, which is induced by bacterial LPS [20]. However, how TLR4 is involved in recognition upon* H. pylori* infection is not really established and much debate is in the literature about this topic. TLR4 and MD2 expression was induced in gastric epithelial cells of infected patients. However, lamina propria mononuclear cells expression of TLR4 and MD2 was similar in uninfected and infected biopsies. In addition,* H. pylori* LPS was not able to induce NF-*κ*B activation in treated AGS epithelial cells but induced NF-*κ*B in THP1 cells. However, MD2 transfection of AGS cells alone was able to regain NF-*κ*B activation in* H. pylori* LPS-treated cells and that was attributed to more cell surface location of TLR4 in this cell line [42]. Maeda and coworkers investigated the involvement of TLR4 and CD14 in NF-*κ*B activation using THP-1 monocytes and MKN45 epithelial cells [43]. They showed that the expression of TLR4 and CD14 was required for activation of NF-*κ*B in monocytes, but not in gastric epithelial cells. Interestingly, NF-*κ*B activation in epithelial cells was dependent on a functional* cag*PAI in* H. pylori*, but not in monocytes [43]. It was also reported that* H. pylori* induced the secretion of IL-12 and IL-10 in mouse macrophages through TLR4/MyD88 signaling [32]. In addition,* H. pylori* primed DCs interaction with allogeneic CD4^+^ T-cells, which resulted in the production of interferon gamma (IFN-*γ*) and IL-17A as well as the induction of transcription factor forkhead-box-protein P3 (FOXP3). This indicates that* H. pylori* induced a mixed T-cell response including Th1, Th17, and Treg. However, blocking of TLR4 signaling resulted in significant reduction of Th1 and Th17 specific cytokines as well as FOXP3 expression [61]. In contrast, other groups have shown that primary gastric epithelial cells do not express TLR4 and were also nonresponsive to LPS [62]. In another study it was demonstrated that infection of AGS and MKN45 cells with* H. pylori* resulted in an upregulation of TLR4; however, adding blocking antibodies towards TLR4 failed to inhibit LPS-induced IL-8 secretion. In line with the abovementioned studies, this study also showed that the whole* H. pylori-*induced IL-8 secretion in epithelial cells and LPS-mediated induction requires CD14 expression [44]. In one of the earlier studies using* H. pylori* LPS it was shown that impaired sensitivity to TLR4 is due to modifications of the lipid A core and this can also be responsible for reduced innate immune response to* H. pylori* [63]. Lipid A prepared from LPS of* H. pylori* strain 206-1 as well as its synthetic form resulted in low endotoxic activity when given orally to C57BL-6 mice, while the corresponding synthetic lipid A from* Escherichia coli* had strong effects as expected. Treatment with* H. pylori* LPS was also shown to decrease IL-8 and TNF-*α* secretion in a human gastric cancer cell line and peripheral blood mononuclear cells (PBMCs), respectively, when compared to synthetic lipid A from* E. coli* [64]. Poor recognition of* H. pylori* LPS was also suggested to depend on the degree of acylation and reduced activation of the immune system is corresponding to a low acylation level of LPS. LPS of* H. pylori* is mostly tetraacylated, while LPS from* E. coli* is hexaacylated [25, 63, 65]. However, exact role of* H. pylori* LPS in TLR recognition is debatable because purified LPS and live bacteria have been shown to result in different pattern of recognition and signaling as reported by different groups. The bioactivity of* H. pylori* LPS to induce TLR4 activation was documented to be 1,000- to 10,000-fold less in comparison to LPS from* E. coli* [63, 66]. This has also been implicated to the variation in fatty acid distribution and phosphorylation status of the lipid A portion of* H. pylori* LPS [67].* H. pylori* LPS was also observed to induce superoxide anions in gastric pit cells of guinea pigs, possibly through TLR4 signaling, and this was also dependent on the phosphorylation status of the lipid A disaccharide backbone [45]. In support of the above data, further studies identified two phosphatases encoded by the* H. pylori* genome, which can modify its lipid A by removal of phosphate groups from the lipid A backbone, and this modification helped to decrease the recognition of this LPS by TLR4 and also the sensitivity to cationic antimicrobial peptides [46, 68]. Interestingly, it was reported that* H. pylori* LPS can promote proliferation and progression of gastric cancer cells via a TLR4-dependent pathway as well as to attenuate cytotoxicity of PBMCs and thus promote cancer formation. The impaired cytotoxicity can be attributed to the attenuated production of Th1 cytokine IL-12, IFN-*γ* in mononuclear cells (MNCs), and perforin levels in natural killer (NK) cells after treatment with* H. pylori* LPS [69]. In addition, the discrepancies found in the literature regarding the activation of TLR4 by LPS might also arise from the different strains and the dosage that is used in these studies, which has already been suggested before [29]. Furthermore, the purity of LPS in different preparations is also a matter of concern because the possible presence of peptidoglycan or lipopeptides in such fractions might alter the activation pattern towards TLR2 instead of TLR4.

### 3.3. TLR5 in* H. pylori *Infection

Flagellin from many bacteria is the only ligand identified for TLR5. The binding of this ligand to TLR5 at the cell surface induces downstream signaling through a MyD88-dependent pathway. The involvement of other adaptor proteins (TRAM/TRIF) dependent downstream signaling pathway has not been reported in the case of TLR5 activation. TLR5 expression was shown to be present in most of the epithelial surfaces studied in humans and mice. This ubiquitous presence of TLR5 shows its importance in the recognition of microbial pathogens at epithelial surfaces [20].* H. pylori* is considered to be an extracellular pathogen, mainly colonizing the human gastric mucosa and surviving there for long periods of time by defeating the efforts of host immune system. The involvement of TLR5 in the recognition and further inflammatory processes was initially proposed to be of importance on establishing a persistent infection of* H. pylori* at the mucosal surface. In one of the first studies,* H. pylori* infection of gastric epithelial cell lines has activated NF-*κ*B through TLR5 and also induced the expression of chemokines such as IL-8, MIP3*α*, and GRO*α*. They have also reported that partially purified* H. pylori* flagellin activated NF-*κ*B, possibly through TLR5 [28]. TLR5-dependent stimulation of IL-8 secretion was activated through the increase in p38 and ERK MAP kinase activity and also showed high activating transcription factor 2 (ATF2) phosphorylation in* H. pylori* infected cells [39]. In contrast, Gewirtz and coworkers reported that infection of AGS cells with Δ*fla*A mutant of* H. pylori* did not significantly reduce the secretion of IL-8 [49]. They have also shown that purified recombinant FlaA of* H. pylori* is a weak inducer of IL-8 expression or p38 activation in gastric epithelial cells. In line with the latter observation, a classical study reported the importance of certain motifs in the flagellin for their recognition through TLR5 [50]. This study used different flagellated and nonflagellated bacteria for analyzing their capacity to activate TLR5. Nonflagellated* Staphylococcus aureus* and flagellated* Bartonella bacilliformis*,* Rhizobium meliloti*,* Campylobacter jejuni*,* H*.* pylori* (strain 26695 and two clinical isolates),* H*.* hepaticus*,* H*.* felis*, and* Wolinella succinogenes* were not recognized by TLR5. The strongest known ligand of TLR5, FliC of* Salmonella enterica*, stimulated TLR5-dependent NF-*κ*B activation. Surprisingly, a chimera of FliC containing the N-terminal D0-D1 domain of* H. pylori* FlaA was completely inactive on stimulating TLR5-dependent NF-*κ*B activation. Further experiments have located the TLR5 stimulatory and nonstimulatory effects within a specific region of the D1 domain of flagellin. Finally, replacement of amino acids 89–96 of FliC with the corresponding amino acids from* H. pylori* FlaA abolished the TLR5 agonist activity of FliC [50]. This study has clearly explained the weak recognition of* H. pylori* flagellin through TLR5 as an important immune evasion process of this long-term colonizing pathogen. However, this study did not completely rule out the possibility of other ligands from* H. pylori* or other nonstimulatory bacteria, because most of their findings were dependent on heat-killed bacteria or purified flagellin. A recent study showed that a chimeric flagellin composed of terminal regions from* E. coli* and the middle region from* H. pylori* folded correctly and was able to activate TLR5. Vaccination using this chimeric recombinant flagellin was able to provide significant protection against* H. pylori* colonization, thus making it an efficient alternative method for vaccinating against other flagellated bacteria that evade TLR5 recognition [51]. We have shown that* H. pylori* significantly induced the upregulation of TLR5 in THP1 and HEK293-TLR5 cells [34].* H. pylori* infection of THP-1 cells induced the secretion of IL-8 and TNF-*α* in a* cag*PAI-dependent manner. In addition, infection of HEK293 cells expressing TLR5 with* H. pylori* induced the phosphorylation of IL-1 receptor-associated kinase 1 (IRAK-1) and inhibitor of kappa B (I*κ*B), and this was required for the activation of NF-*κ*B. However, induced expression of transfected TLR5 in HEK293 cells shifted* cag*PAI dependent to* cag*PAI independent proinflammatory signaling for the secretion of IL-8 and TNF-*α* [34]. In addition, TLR5 mRNA expression level was upregulated in the gastric epithelial cell line GES-1 during infection with spiral-shaped* H. pylori*, but not by the corresponding coccoid form [52]. In contrast, a study using gastric biopsies has ruled out the induction of TLR5 expression in* H. pylori* infection [47]. Moreover, TLR5 expression in the gastric epithelium of chronic active* H. pylori* gastritis was localized at the basolateral sides of the cells without detectable expression at the apical side, but was homogeneously distributed in the gastric epithelium with intestinal metaplasia and dysplasia as well as in gastric carcinoma cells [48, 53]. From the above data, we can advocate an involvement of TLR5 in* H. pylori* infection for activation of proinflammatory changes, but the involved mechanism is yet unknown.

### 3.4. TLR8 and TLR9 in* H. pylori *Infection

The nucleic acid sensing TLRs are widely studied in their roles in the recognition and induction of successive immune responses against different viruses. However, the literature for microbial RNA sensing various TLRs (TLR3, TLR7, and TLR8) is limited for bacterial infections. These receptors are localized on subcellular structures and induce signaling through a MyD88-dependent pathway except for TLR3, which was reported to mediate signaling by the adaptor protein TRIF [70]. Interestingly, it has been reported that* H. pylori* phagocytosis by THP1 cells induced the expression of two functional TLR8 isoforms and TNF-*α* secretion.* H. pylori* phagocytosis significantly activated the sensing of synthetic TLR8 ligands in those cells for the secretion of cytokines. This emphasizes an active role of TLR8 in* H. pylori* recognition and immune responses [54]. In addition, it was shown that purified* H. pylori* RNA induced a strong IL-6 and IL-12 response in mouse DCs and this was dependent on MyD88, but not on TLR9 and TLR7 [30]. TLR9 is known to sense unmethylated CpG DNAs of bacterial, viral, or synthetic origin and signal through MyD88-dependent pathway.* H. pylori* clinical isolates induced cyclooxygenase 2 (COX2) expression through MAP kinase signaling in epithelial cells and increased the cellular invasion properties, which is partially dependent on TLR9 [40, 41]. In addition,* H. pylori* DNA was shown to induce a more pronounced invasion of a gastric cancer cell line reported through an* in vitro* invasion assay [71]. Rad and coworkers also showed a TLR9 dependent recognition of* H. pylori* DNA and induction of proinflammatory cytokines in mouse DCs [30]. TLR9 expression was predominantly localized at the gastric surface epithelium; however, chronic active gastritis in* H. pylori* infection changed the expression exclusively to the basolateral side and the incoming neutrophils also showed high TLR9 expression [48]. Similarly, infection of isolated neutrophils has also shown an elevated TLR9 expression in a* cag*PAI-independent manner [55]. It has also been demonstrated that TLR9 expression increased in epithelial and immune cells infiltrating to the lamina propria and submucosa of* H. pylori* infected mice. TLR9^−/−^ mice showed the same level of* H. pylori* colonization; however, the myeloperoxidase (MPO) activity and mRNA expression of TNF-*α* and IFN-*γ* were increased in the gastric tissue during the initial phase of infection. In addition, type-I IFN such as IFN-*α* and IFN-*β* mRNA expression was significantly reduced in the TLR9^−/−^ mice during infection. Moreover, recombinant IFN-*α* administration significantly reduced MPO activity and mRNA expression of TNF-*α* and IFN-*γ* in these infected TLR9^−/−^ mice. These data indicate the anti-inflammatory role of TLR9 during the early phase of* H. pylori* induced gastritis and that could be possibly through the production of type-I IFN [56]. Apart from the latter study, it has also been reported that genomic* H. pylori* DNA contained more immune-regulatory sequences (IRS) and was found to be a weak inducer of type-I IFN or IL-12 secretion from mouse DCs and also suppressed the induction of these cytokines by* E. coli* DNA. In concurrence with the anti-inflammatory response reported in the above studies,* H. pylori* DNA administration before the induction of dextran sodium sulfate (DSS) mediated colitis significantly ameliorated the severity of colitis. This also supports the hypothesis of inverse relationship between* H. pylori* and inflammatory bowel diseases [72].* H. pylori* genome specific IRS 5′-TTTAGGG-3′ with other IRSs might constitute the inhibitory effect of* H. pylori* genomic DNA through TLR9 signaling pathway [73]. Taken together, the above studies indicate that* H. pylori* nucleic acids are recognized by TLRs and inducing both pro- and anti-inflammatory responses during infection. These contrasting observations must be studied in detail to understand differential factors influencing shifting of responses and to see if this is helping the host or bacteria in the hostile interaction.

## 4. Role of TLR Gene Polymorphisms in* H. pylori* Infection

It has been noted that the sequence of* TLR* genes can slightly vary between patients. Hundreds of small nucleotide polymorphisms (SNPs) have been identified in various* TLR* genes, but the functional consequences of the majority of SNPs remain unknown. Many associations have been reported between TLR polymorphisms and infectious diseases or cancers. In the case of* H. pylori* infection, TLR polymorphisms have been specifically implicated to enhance the susceptibility for infection (TLR1) and also the risk of developing* H. pylori*-induced gastric cancer (TLR4).

### 4.1. Susceptibility to* H. pylori* Infection

Several small case control studies have addressed the role of genetic polymorphisms in the risk of* H. pylori* infection, but only one Genome Wide Association Study (GWAS) has so far been published [74]. Mayerle and colleagues reported two independent GWAS studies from two independent population-based cohorts from north-eastern Germany (Study of Health in Pomerania) and Netherlands (Rotterdam Study) [74]. Fecal* H. pylori* antigen testing was used to determine the presence of infection in these individuals. GWAS meta-analysis identified 2 genome-wide significant loci in terms of their association to* H. pylori* seropositivity, namely, the* TLR* locus on chromosome 4p14 and the* FCGR2A* locus on chromosome 1q23.3. The lead SNP on the* TLR* locus with the lowest *P* value was rs10004195 (OR = 0.70, 95% CI = 0.65–0.76), closely followed by rs4833095 (OR = 0.70, 95% CI = 0.65–0.76). Three different TLRs are located along the 4p14 region: TLR1, TLR6, and TLR10. In an additional study conducted on 1,763 participants from both cohorts, analysis of whole blood RNA gene expression profiling showed that among the three* TLR* genes, only TLR1 was differentially expressed in relation to the rs10004195 genotype (in the presence of the rs10004195-A allele [beta = −0.23, 95% CI = −0.34 to −0.11]). Furthermore, analysis of TLR1, TLR6, and TLR10 mRNA amounts also showed that there was a specific and genotype-independent transcriptional upregulation of TLR1 in the presence of* H. pylori*. These results imply that the increase in TLR1 mRNA expression as a result of the rs10004195 SNP is strongly associated with an increased risk of* H. pylori* seropositivity.

The mechanism for the relationship between increased TLR1 expression and a higher* H. pylori* seroprevalence remains unexplained. However, TLR1 has been shown to interact with TLR2 to form a heterodimer [75], which is responsible for the initiation of cellular downstream signaling in response to the recognition of triacylated lipopeptides from the Gram-negative bacterial cell wall [76]. This is particularly relevant to* H. pylori* infection as triacylated lipopeptides can be found in the structure of* H. pylori* lipid A, allowing it to be recognized by the TLR1-TLR2 complex. It has been suggested that the resulting activation of the immune cascade could reduce the anti-inflammatory response of the host against* H. pylori*, thus allowing persistent infection [76]. Another explanation proposed recently is that the SNP at the* TLR1* gene causes less effective anti-inflammatory signaling initiated by the TLR1-TLR2 complex in response to the presence of* H. pylori*, thus increasing the risk of persistent infection [77].

### 4.2. Risk of TLR SNPs in* H. pylori*-Induced Gastric Cancer

Hold and coworkers reported that the* TLR4*+896A>G polymorphism was associated with risk of gastric cancer and its precursors [78].* TLR4*+896G carriers had an 11-fold (95% confidence interval [CI], 2.5–48) increased odds ratio (OR) for hypochlorhydria and also had significantly more severe gastric atrophy and inflammation. Seventeen percent of gastric carcinoma patients in the initial study and 15% of the noncardia gastric carcinoma patients in the replication study had 1 or 2 TLR4 variant alleles* versus* 8% of both control populations (combined OR = 2.3; 95% CI = 1.6–3.4) [78]. In a case-control study and meta-analysis, Castaño-Rodríguez and coworkers reported that the TLR signaling pathway was implicated in gastric carcinogenesis, with* TLR4* Asp299Gly and* TLR2* −196 to −174 del showing associations with gastric cancer in an ethnic-specific manner [79]. Although not fully understood, these observations highlight the importance of TLR2 and TLR4 in gastric disease development.

## 5. Conclusions and Outlook

*H. pylori* is one of the most successful bacterial pathogens infecting about half of the human world population and is responsible for a considerable global health burden, including peptic ulcer disease and gastric cancer. Studies of host-bacterial interactions using their fundamental virulence-associated factors have provided us with remarkable insights into* H. pylori* biology. Here we have reviewed the interference of a multitude of bacterial factors with five TLRs (TLR2, TLR4, TLR5, TLR8, and TLR9). The current data suggest a model in which* H. pylori* can interact with or evade these TLRs (Figure 1). In addition, we have reviewed our current knowledge on the bacterial factors including HSP-60, NapA, LPS, DNA, or RNA and how they may target TLRs and downstream signaling cascades. It can be therefore assumed that there is a highly dynamic system of extensive crosstalk between TLRs and their bacterial ligands to make up a scenario of complex host cellular processes leading to persistent colonization and chronic pathogenicity. However, there are some discrepancies with regard to corresponding* H. pylori* ligands or new molecular patterns to induce different signaling pathways for the production of mediators of the host immune system as discussed here. In the future, it will therefore be important to solve some of the conflictive reports discussed above. For example, the bacterial factors activating TLR2 and TLR4. In addition, it will be fundamental to unravel if new* H. pylori* factors can target TLRs for inducing downstream signaling. Finally, SNPs were found in various TLRs and they are known to crucially influence the clinical outcome of* H. pylori* infections. These factors therefore appear to play a key role in the pathophysiology of gastric disease development and their importance should be investigated in animal models which mimic human gastric neoplasia. It can be expected that more genetic polymorphisms both in the host and in* H. pylori* will be uncovered with advancing technologies, so that there is every prospect of defining full genetic risk profiles in near future. This will also aid in improving the current testing and treatment strategies of* H. pylori* infections. Thus, it appears that* H. pylori*-TLR receptor interactions will continue to be a fascinating and rewarding research topic in future studies.

## Figures and Tables

**Table 1:** Targeted TLR receptors with proposed role during infection with *H. pylori*.

TLR receptor	Host cells and mouse lines used	*H. pylori* strain(s) used	Applied methods	Proposed role during infection	References
TLR2	AGS, HEK293, MKN45, CHO	26695, LC11, 98, 99	RT-PCR, LRGA	NF-*κ*B activation and chemokine expression	[28]
	HEK293	26695	ELISA, IP, WB, SPIA	MAPK activation and chemokine expression	[39]
	HEK293, PBMCs, primary human monocytes and macrophages, PECs	SS1, 43504, Astra 244	ELISA, RT-PCR, cDNA-MA	Intact bacteria activate TLR-2, while LPS activates TLR-4	[29]
	mBMDCs	SS1, J99, TX30, B128	ELISA, RT-PCR, cDNA-MA, FACS, LRGA, KD	Activates TLRs to induce production of pro- and anti-inflammatory cytokines	[30]
	MKN28, MKN45, HEK293, T24, THP1	Clinical strains	ELISA, FACS, RT-PCR, LRGA, WB	Highly purified LPS is a weak agonist, activates NF-*κ*B through TLR-2/1 heterodimer	[35]
	KATO III	43504	ELISA, ABB, RT-PCR, NB, WB, TFA, ICC, RPT	HpHSP60 is a ligand, activates NF-*κ*B and chemokine expression in epithelial cells	[36]
	NOMO1, U937	43504	ELISA, ABB, TFA, RT-PCR, siRNA, SPIA, FACS, WB, RPT	HpHSP60 is a ligand, activates MAPK and chemokine expression in monocytes	[37]
	PBMCs, primary human monocytes, neutrophils and T-cells	n.p.	ELISA, RT-PCR, ELISPOT, NF-*κ*BRA, FACS, TCA, CRA, RPT	HpNAP activates proinflammatory cytokine expression and T-cell responses	[38]
	AGS, MKN45	Clinical strains	EMSA, RT-PCR, WB, IFM, LGRA, IP, KA	NF-*κ*B activation and COX-2 overexpression	[40]
	AGS, MKN45, HUVEC	Clinical strains	ELISA, DAPA, EMSA, WB, ChIP, RT-PCR, LGRA, MGTA	COX-2 induction and increased cell invasion and angiogenesis	[41]

TLR4	AGS, HEK293, MKN45, CHO	26695, LC11, 98, 99	RT-PCR, LRGA	NF-*κ*B activation and chemokine expression	[28]
	HEK293, PBMCs, primary human monocytes and macrophages, PECs	SS1, 43504, Astra 244	ELISA, RT-PCR, cDNA-MA	Intact bacteria activate TLR-2, while LPS activates TLR-4	[29]
	AGS, MKN-7, MKN-28, MKN-45, THP-1	43504, TN583, clinical strains	LCM, IHC, RT-PCR, FACS, LGRA	NF-*κ*B activation and chemokine expression	[42]
	MKN45, TMK1, J774A.1, THP1, PECs	TN2	ELISA, ABB, TLR-SA, RT-PCR, EMSA, RPA, IP, WB, cDNA-MA	NF-*κ*B activation is *cag*PAI-dependent in epithelial cells, but *cag*PAI-independent in monocytes/macrophages	[43]
	AGS, MKN45, CHO, T84, THP1	LC11, LC20	ELISA, RT-PCR, WB, FACS, IFM, SEM	Upregulation of TLR expression and chemokine secretion	[44]
	Gastric mucosal cells	11637, 11638, clinical strains	RT-PCR, SB, WB, NB	Upregulation of superoxides in gastric pit cells	[45]
	HEK293 C57BL/6J mice *in vivo *	J99, B128, X47	FBA, MS, PA, TLR-SA, MCA, NF-*κ*BRA, FM	Dephosphorylation of lipid-A reduces recognition and increases colonization	[46]
	AGS, gastric biopsies	J99, clinical strains	RT-PCR	TLR expression is not affected in gastric biopsies of infected patients	[47]
	Gastric biopsies	Clinical strains	ICH, IFM, CSLM	Shifting of subcellular localization of TLRs	[48]

TLR5	AGS, HEK293, MKN45, CHO	26695, LC11, 98, 99	RT-PCR, LRGA	NF-*κ*B activation and chemokine expression	[28]
	HEK293	26695	ELISA, IP, WB, SPIA	MAPK activation and chemokine expression	[39]
	AGS, T84, MDCK	49503	ELISA, WB, RPT,	Flagellin evades TLR5 recognition	[49]
	CHO K1	G27, clinical strains	LRGA, WB, RPT, BIT, MA	Flagellin evades TLR5 recognition	[50]
	HEK293 BALB/c mice *in vivo *	SS1S	ELISA, CD, LRGA, RT-PCR, WB, RPT	Chimeric flagellin can activate immune responses	[51]
	HEK293, THP1	P1, P12, P310, 26695	ASPAB, ELISA, IFM, RT-PCR, TLR-SA, LRGA, WB	*cag*PAI status can change TLR activated production of cytokine/chemokine	[34]
	GES1	26695	cDNA-MA, FACS, RT-PCR, SEM	Spiral and coccoid forms can influence TLR expression	[52]
	AGS, gastric biopsies	J99, clinical strains	RT-PCR	TLR expression is not affected in gastric biopsies of infected patients	[47]
	Gastric biopsies	Clinical strains	ICH, IFM, CSLM	Shifting of subcellular localization of TLRs	[48]
	Gastric biopsies	Clinical strains	ICH, IFM, CSLM	Shifting of subcellular localization of TLRs	[53]

TLR8	PBMCs, primary human monocytes, HeLa, HEK293, HEK293T, HEK293FT, THP1	251, B128	ELISA, RT-PCR, LGRA, CSLM, BIT, FACS	Bacterial phagocytosis increases TLRs activation and cytokine secretion	[54]

TLR9	mBMDCs	SS1, J99, TX30, B128	ELISA, RT-PCR, cDNA-MA, FACS, LRGA, KD	Activates TLRs to induce production of proinflammatory cytokines	[30]
	AGS, MKN45, HUVEC	Clinical strains	ELISA, DAPA, EMSA, WB, ChIP, RT-PCR, LGRA, MGTA	COX-2 induction and increased cell invasion and angiogenesis	[41]
	AGS, MKN45	Clinical strains	EMSA, RT-PCR, WB, IFM, LGRA, IP, KA	NF-*κ*B activation and COX-2 overexpression	[40]
	Gastric biopsies	Clinical strains	ICH, IFM, CSLM	Shifting of subcellular localization of TLRs	[48, 53]
	Primary human neutrophils	26695, G27, 8822, clinical strains	ELISA, FACS	*cag*PAI dependent production of pro- and anti-inflammatory cytokines	[55]
	Gastric tissue, C57BL/6J mice	SS1	IHC, RT-PCR, MPA, CSLM	Type-I interferon mediated anti-inflammatory response at early phase infection	[56]

AB: antibody; ABB: antibody blocking; ASPAB: activation specific phospho antibodies; BIT: bioinformatic tools; *cag*PAI: cytotoxin-associated genes pathogenicity island; CD: circular dichroism; cDNA-MA: cDNA micro/macroarray; ChIP: chromatin immunoprecipitation; COX-2: cyclooxygenase-2; CRA: chromium release assay; CSLM: confocal laser scanning microscopy; DAPA: DNA affinity protein binding assay; ELISA: enzyme-linked immunosorbent assay; ELISPOT: enzyme-linked immunospot; EMSA: electrophoretic mobility shift assay; FACS: fluorescence-activated cell sorting; FBA: fluorescent binding assay; FM: fluorescence microscopy; HPA: histopathological analysis; HSP: heat shock protein; IFM: immunofluorescence microscopy; ICC: immunocytochemistry; IHC: immunohistochemistry; IP: immunoprecipitation; KA: kinase assay; KD: knockdown of genes; LCM: laser capture microdissection; LRGA: luciferase reporter gene assay; MAPK: mitogen-activated protein kinases; MA: motility assay; mBMDCs: mouse bone marrow derived DCs; MCA: mouse colonization assay; MGTA: matrigel tube formation assay; MPA: myeloperoxidase activity assay; MS: mass spectrometry; NAP: neutrophil activating protein; NB: northern blotting; NF-*κ*B: nuclear factor kappa B; NF-*κ*BRA: nuclear factor kappa B reporter assay; n.p.: not provided; PA: phosphatase assay; PBMCs: peripheral blood mononuclear cells; PECs: peritoneal exudate cells; RPA: RNAse protection assay; RPT: recombinant protein techniques; RT-PCR: real-time/reverse transcriptase PCR; SB: southern blotting; SEM: scanning electron microscopy; siRNA: small interfering RNA; SPIA: signaling pathway inhibitor assay; TCA: T-cell clonal assays; TFA: transcription factor assay; TLR: Toll-like receptor; TLR-SA: TLR signaling assay; WB: western blotting.

## Abbreviations Used

AP-1: Activator protein 1
APC: Antigen presenting cell
IFN: Interferon
IKKs: I*κ*B kinases
IRF3: Interferon regulatory factor 3
Lipid A: A component of LPS
LPS: Lipopolysaccharide
MAPK: Mitogen-activated protein kinase
MyD88: Myeloid differentiation primary response gene 88
NF-*κ*B: Nuclear factor kappa B
PAMP: Pathogen-associated molecular pattern
TBK1: Tank-binding kinase 1
TIR: Toll/interleukin-1 receptor domain
TRAF3/6: TNF receptor associated factor 3/6
TRIF: TIR-domain-containing adapter-inducing interferon-*β*.
